# Early vascular damage in retinal microcirculation in arterial hypertension: the Czech post-MONICA study

**DOI:** 10.1097/HJH.0000000000003637

**Published:** 2023-12-13

**Authors:** Renata Cífková, Joanna M. Harazny, Jan Bruthans, Peter Wohlfahrt, Alena Hrubeš Krajčoviechová, Věra Lánská, Július Gelžinský, Markéta Mateřánková, Štěpán Mareš, Jan Filipovský, Otto Mayer, Roland E. Schmieder

**Affiliations:** a Center for Cardiovascular Prevention, Charles University in Prague, First Faculty of Medicine and Thomayer University Hospital; b Department of Medicine II, Charles University in Prague, First Faculty of Medicine, Prague, Czech Republic; c Department of Nephrology and Hypertension, University Hospital Erlangen, Friedrich-Alexander- University, Erlangen-Nürnberg, Germany; d Department of Human Physiology and Pathophysiology, University of Warmia and Mazury, Olsztyn, Poland; e 2nd Department of Internal Medicine, Faculty of Medicine, Charles University, Pilsen; f Medical Statistics Unit, Institute for Clinical and Experimental Medicine, Prague, Czech Republic

**Keywords:** Czech post-MONICA study, juxtapapillary retinal capillary blood flow, lumen diameter, retinal vascular resistance, scanning laser doppler flowmetry, vessel diameter, wall cross-sectional area, wall thickness, wall-to-lumen ratio

## Abstract

Retinal microcirculation reflects retinal perfusion abnormalities and retinal arterial structural changes at relatively early stages of various cardiovascular diseases. Wall-to-lumen ratio (WLR) may represent the earliest step in hypertension-mediated organ damage.

Our objective was to compare functional and structural parameters of retinal microcirculation in a randomly selected urban population sample, in hypertensive and normotensive individuals.

**Design and method:**

A total of 398 randomly selected individuals from an urban population aged 25–65 years, residing in Pilsen, Czech Republic, were screened for major cardiovascular risk factors. Retinal microcirculation was assessed using scanning laser Doppler flowmetry, with data evaluable in 343 patients. Complete data were available for 342 individuals divided into four groups based on blood pressure and control status of hypertension: normotensive individuals (*n* = 213), treated controlled hypertensive individuals (*n* = 30), treated uncontrolled hypertensive individuals (*n* = 26), and newly detected/untreated hypertensive individuals (*n* = 73).

**Results:**

There was a tendency to higher wall thickness in treated but uncontrolled hypertensive patients (compared to normotensive and treated controlled hypertensive individuals). WLR was significantly increased in treated but uncontrolled hypertensive patients as well as in individuals with newly detected thus untreated hypertension or in patients with known but untreated hypertension. There was no difference in WLR in treated, controlled hypertensive patients compared with normotensive individuals.

**Conclusion:**

Our results show that an increased WLR, reflecting early vascular damage, was found in newly detected individuals with hypertension and in untreated hypertensive patients, reflecting early hypertension-mediated vascular damage. Early initiation of hypertension treatment may be warranted.

## INTRODUCTION

Hypertension is the most common cardiovascular disorder affecting 20–50% of the adult population worldwide. In 2019, high SBP was identified as the leading risk factor for global attributable deaths [1].

Increased peripheral resistance is the hallmark of hypertension and is caused by structural and functional changes in the resistance vasculature. The primary site of increased vascular resistance is the microcirculation, consisting of small arteries (diameter 100–300 μm), arterioles (diameter <100 μm), and the capillary network (diameter approx. 7 μm) [2]. In the early stages of the development of hypertension, there is usually constriction of small arteries and arterioles due to various nervous or endocrine and autocrine mechanisms. These functional changes may be followed by inward hypertrophy of small arteries and arterioles as well as gradual rarefaction of arterioles and capillaries. The consequences of structural alterations include the development of hypertension with possible perfusion abnormalities in various organs, increasing the risk of cardiovascular morbidity and mortality [3,4].

The clinical evaluation of a hypertensive patient should include a search for hypertension-mediated organ damage (HMOD) or existing cardiovascular, cerebrovascular, or renal disease [5]. Hypertensive retinopathy was first described in the 19th century and has, for a long time, been considered a marker of the degree of target organ damage in hypertension. The current guidelines recommend fundoscopy in patients with grade 2 or 3 hypertension, and in all hypertensive patients with diabetes. Retinal hemorrhages, microaneurysms, hard exudates, cotton wool spots, and papilledema are indicative of severe hypertensive retinopathy, are highly reproducible and are associated with a high risk of mortality [6,7]. On the contrary, arteriolar narrowing and arteriovenous nicking, typical in the early stages of hypertensive retinopathy, have less predictive value [8] and also lower reproducibility [9].

Retinal microcirculation reflects retinal perfusion abnormalities and retinal arterial structural changes at relatively early stages of various cardiovascular diseases. The wall-to-lumen ratio (WLR) of retinal arterioles may possibly represent the earliest step in HMOD. Moreover, a recent review [10] suggested that the retinal microvasculature can provide essential data about concurrent cardiac disease status and predict a future risk of events.

New technologies such as scanning laser Doppler flowmetry (SLDF) have been developed to assess retinal microcirculation by measuring flow and structure and quantifying the WLR of retinal arterioles [11,12], with diameters analyzed off-line using automatic full field perfusion imaging analysis [13].

Our objective was to compare functional and structural parameters of retinal microcirculation in a randomly selected urban population sample, in hypertensive treated and untreated as well as normotensive individuals.

## MATERIALS AND METHODS

### Study population

A total of 398 randomly selected individuals from an urban population aged 25–65 years, resident of Pilsen, Czech Republic, were screened for major cardiovascular risk factors within the Czech post-MONICA study from October to November 2017 [14]. A 1% population sample, stratified by age and sex, was obtained from registers of five major health insurance companies in the Czech Republic, covering 85% of the total population. Health insurance is mandatory for all individuals living in the Czech Republic and is paid for by the employer/employee or by the government for children, the retired and unemployed.

The study was approved by the Ethics Committee of the Institute for Clinical and Experimental Medicine and Thomayer University Hospital, Prague, Czech Republic. All participants signed informed consent.

### Screening examination

The methods used were described in detail elsewhere [14]. In brief, the examination procedure started with a questionnaire, which was completed by a physician. Currently prescribed drugs were recorded and verified (if possible) against drug containers. Height and body weight measurements were taken, blood pressure (BP) was consistently measured on the right arm (supported at heart level), in the sitting position, after at least a 5-min rest. Three consecutive BP measurements were obtained using standard mercury sphygmomanometers (Baumanometer; W.A. Baum Co. Inc., New York, New York, USA) and correctly sized cuffs. Blood pressure values were recorded to the nearest 2 mmHg. The mean value of the last two readings was used for the analyses. Additionally, unattended automated office BP (AOBP) was taken using an oscillometric device (BpTRU, model BP300, BpTRU Medical Devices Inc., Coquitlam, British Columbia, Canada). The first BP reading (validating measurement) was automatically deleted and the mean of the following five consecutive readings was used for the analysis. Aortic stiffness was assessed using carotid–femoral pulse wave velocity (PWV) employing the SphygmoCor device (AtCor Medical Ltd, West Ryde, New South Wales, Australia) with the examined individuals in the recumbent position [15] following a previously used standardized protocol [15]. The distance between the carotid and femoral sites was directly measured on the body surface and multiplied by 0.8 (D), PWV was calculated as D (m)/Δt (s).

A gentle venous blood sampling was performed in the sitting position after at least a 12-h fast. The obtained samples were centrifuged at 1500 *g* and frozen thereafter.

### Assessment of retinal microcirculation

Retinal microcirculation was assessed using scanning laser Doppler flowmetry (SLDF; Heidelberg Retina Flowmeter, Heidelberg Engineering GmbH, Germany) at a wavelength of 670 nm, to determine juxtapapillary retinal capillary blood flow (JRCF), vessel diameter, lumen diameter, wall thickness, WLR, wall cross-sectional area (WCSA), and retinal vascular resistance (RVR). All measurements were obtained from retinal arterioles of the right eye, in the juxtapapillary area, 2–3 mm temporal superior of the optic nerve, in individuals sitting in a dark room. After 15 min of rest and without the application of mydriatic agents, the measurements were taken by experienced investigators.

The same experienced investigator (J.H.) analyzed all images offline using AFFPIA Version 4.10 [13,16]. This software automatically eliminates vessel diameter more than 20 μ. Vessel diameter was measured in reflection images and lumen diameter in perfusion images. Wall thickness was calculated as VD - LD/2 and WCSA using the formula (π/4) x (VD^2^ - LD^2^), whereas WLR was calculated using the formula (VD - LD)/LD [11]. The mean value of three images was used for the analyses.

### Laboratory analyses

Lipid parameters were analyzed in the Lipid Laboratory of the Institute for Clinical and Experimental Medicine, Prague, Czech Republic, employing a fully automated enzymatic method (COBAS MIRA S analyzer, Roche Diagnostics, Basel, Switzerland), along with enzymatic kits produced by the same manufacturer. LDL-cholesterol was calculated using the Friedewald formula. Serum and urine creatinine were analyzed using an enzymatic assay (Hitachi 902 autoanalyzer, Roche Diagnostics, Basel, Switzerland). Glycosylated hemoglobin (HbA1c) was assessed using HLCP method (D-10 analyzer, Bio-Rad, Hercules, California, USA), whereas urine albumin was determined using an immunoturbidimetry (COBAS MIRA S analyzer, Roche Diagnostics, Basel, Switzerland); both these analyses were performed by the Department of Clinical Biochemistry at the Thomayer University Hospital, Prague, Czech Republic. The accuracy of these analyses was continuously monitored and tested, with external quality control of lipid parameters performed by the Centers for Disease Control and Prevention (Atlanta, Georgia, USA).

### Definition of hypertension and major cardiovascular risk factors

Hypertension was defined as a mean SBP at least 140 mmHg, and/or a mean DBP at least 90 mmHg (the mean of the second and third readings), or current treatment with antihypertensive drugs. The following definition was used for dyslipidemia: total cholesterol at least 5 mmol/l (∼190 mg/dl) or HDL-cholesterol less than 1 mmol/l (∼40 mg/dl) in men and less than 1.2 mmol/l (∼45 mg/dl) in women or use of lipid-lowering drugs. Diabetes was defined as HbA1c at least 48 mmol/l or treatment for diabetes (oral hypoglycemic drugs or insulin).

### Statistical analysis

Continuous variables were described by mean, standard deviation, median, 25% and 75% percentiles, discrete ones by absolute and relative frequencies.

Gaussian distribution was tested using the Shapiro–Wilk test. The analysis of variance (ANOVA), Kruskal–Wallis test, and chi-square test were used for comparison of the four groups (for definition, see the first paragraph of the Results section). Adjustment for age, sex, HbA1c, and triglycerides was performed by analysis of covariance (ANCOVA). Method of contrast was used for the detection of significant pairs. All analyses were performed using statistical software JMP 16.2.0 (2020–2021, SAS Institute Inc., Cary, NC, USA). All tests were two-sided and *P* value less than 0.05 was considered statistically significant.

## RESULTS

### Basic characteristics of the study population

Out of the 398 screened individuals, evaluable data of retinal microcirculation were obtained from 343 of them. Of this number, complete data were available for 342 individuals who were divided into the following four groups based on office BP (the mean of the second and third readings) and treatment of hypertension: normotensive individuals, treated controlled hypertensive individuals, treated uncontrolled hypertensive individuals, and newly detected/untreated hypertensive individuals. Newly detected cases were those who had never been told by healthcare professionals that they had hypertension and had never been prescribed antihypertensive drugs. On the basis of this definition, we found 73 individuals; 57 who were newly detected during the screening examination and 16 who were aware of having hypertension but were not taking antihypertensive medication.

Descriptive statistics of the four groups are presented in Table 1.

**TABLE 1:** Basic characteristics of the study groups

	Normotensives	Treated hypertensives, controlled	Treated hypertensives, uncontrolled	Newly detected/ untreated hypertensives	*P*	NT vs. Treated HT ctrl *P* (Tukey-Kramer HSD)	NT vs. Treated HT unctrl *P*	NT vs. newly detected/ untreated HT *P*	Treated HT ctrl vs. treated HT unctrl *P*	Treated HT ctrl vs. newly detected/ untreated HT *P*	Treated HT unctrl vs. newly detected/ untreated HT; *P*
Numbers	213	30	26	73							
Men/women	85/128	17/13	17/9	43/30	0.0045	n.s.	n.s.	0.0372	n.s.	n.s.	n.s.
Age (years)	44.6 ± 10.8	55.1 ± 10.1	56.7 ± 7.7	51.1 ± 9.4	< 0.0001	< 0.0001	< 0.0001	< 0.0001	n.s.	n.s.	n.s.
BMI (kg/m^2^)	26.4 ± 4.9	31.2 ± 5.0	31.3 ± 6.1	29.4 ± 4.9	< 0.0001	< 0.0001	< 0.0001	< 0.0001	n.s.	n.s.	n.s.
SBP (mmHg)	118.8 ± 11.4	124.8 ± 9.4	148.4 ± 9.6	145.7 ± 13.3	NA						
DBP (mmHg)	77.1 ± 7.6	80.3 ± 6.7	90.9 ± 7.5	93.1 ± 6.6	NA						
Automated office SBP (mmHg)	109.5 ± 11.7	115.9 ± 8.5	135.0 ± 16.3	132.8 ± 14.8	< 0.0001	0.0510	< 0.0001	< 0.0001	< 0.0001	< 0.0001	n.s.
Automated office DBP (mmHg)	72.3 ± 7.6	75.6 ± 7.0	83.2 ± 9.2	85.4 ± 8.0	< 0.0001	n.s.	< 0.0001	< 0.0001	0.0015	< 0.0001	n.s.
Total cholesterol (mmol/l)	5.12 ± 0.95	4.87 ± 0.85	5.41 ± 0.91	5.40 ± 0.96	0.0250	n.s.	n.s.	n.s.	n.s.	0.0499	n.s.
Triglycerides (mmol/l)^a^	0.92 (0.69–1.33)	1.10 (0.76–1.64)	1.46 (1.08–1.77)	1.14 (0.72–1.82)	0.0004	n.s.	0.0007	n.s.	n.s.	n.s.	n.s.
HDL-cholesterol (mmol/l)	1.56 ± 0.38	1.51 ± 0.48	1.33 ± 0.31	1.49 ± 0.46	0.0405	n.s.	0.0347	n.s.	n.s.	n.s.	n.s.
LDL-cholesterol (mmol/l)	3.07 ± 0.85	2.81 ± 0.83	3.40 ± 0.82	3.32 ± 0.86	0.0141	n.s.	n.s.	n.s.	n.s.	0.0428	n.s.
HbA_1c_ (mmol/mol)	34.0 ± 7.5	37.5 ± 7.7	37.9 ± 7.8	34.8 ± 4.7	0.0008	0.0151	0.0092	n.s.	n.s.	n.s.	n.s.
Serum uric acid (μmol/l)	305 ± 82	352 ± 74	356 ± 81	343 ± 75	< 0.0001	0.0173	0.132	0.0032	n.s.	n.s.	n.s.
Urinary albumin/creatinine (g/mol)^a^	0.40 (0.26–1.15)	0.29 (0.23–1.23)	0.55 (0.43–1.08)	0.56 (0.26–1.44)	n.s.	n.s.	n.s.	n.s.	n.s.	n.s.	n.s.
eGFR (ml/s/1.73 m^2^)	97.7 ± 13.9	88.1 ± 13.2	92.4 ± 12.5	92.6 ± 11.6	0.0003	0.0017	n.s.	0.0281	n.s.	n.s.	n.s.
Smoking					n.s.	n.s.	n.s.	n.s.	n.s.	n.s.	n.s.
Current smoker (%)	46 (21.6)	7 (23.3)	6 (24.0)	14 (19.4)							
Never-smoker (%)	125 (58.7)	17 (56.7)	12 (48.0)	41 (56.9)							
Ex-smoker (%)	35 (16.4)	6 (20.0)	7 (28.0)	16 (22.2)							
Occasional smoker (%)	7 (3.3)	0	0	1 (1.4)							
Dyslipidemia (%)	133 (63.3)	23 (79.3)	24 (96.0)	62 (86.1)	< 0.001	n.s.	0.0026	0.0009	n.s.	n.s.	n.s.
Diabetes (%)	7 (3.3)	2 (6.9)	5 (19.2)	4 (5.6)	0.0405	n.s.	0.0433	n.s.	n.s.	n.s.	n.s.
C-f pulse wave velocity (m/s)	7.6 ± 1.5	8.1 ± 2.8	9.7 ± 1.5	8.9 ± 1.6	< 0.0001	n.s.	< 0.0001	< 0.0001	0.0041	n.s.	n.s.

Values are expressed as mean ± SD and absolute and relative frequencies.

a Values are expressed as median (interquartile range, IQR). C-f, carotid-femoral; ctrl, controlled; eGFR, estimated glomerular filtration rate; HbA1c, glycated hemoglobin; HDL, high-density; HT, hypertensive individuals; LDL, low-density; NT, normotensive individuals; unctrl, uncontrolled.

There were 56 individuals treated for hypertension, 21 (40.4%) of them with monotherapy, 10 (19.2%) individuals with a combination of two drugs, and 21 (40.4%) reported a combination of three or more drugs. Detailed information on antihypertensive drug treatment was missing in four individuals. There was no significant difference in the distribution of monotherapy or combination therapy between controlled and uncontrolled hypertensive patients. The most frequently prescribed class of drugs were ACE inhibitors (63.5%) followed by calcium channel blockers (44.2%), diuretics (30.8%), and betablockers (28.8%). Angiotensin receptor blockers (ARBs) were reported by only 11 individuals (21.2%).

Automated office BP was, on average, 10/5 mmHg lower than the office BP values. Treated but uncontrolled hypertensive individuals showed the highest BP values by both measurement techniques and were also the oldest subgroup with the greatest proportion of individuals with dyslipidemia and diabetes. They also had the highest c-f pulse wave velocity. Newly detected/untreated hypertensive individuals displayed similar values in most of the parameters without any significant differences. We also performed another analysis wherein the study population was divided into the same four groups, but the division was based on the mean of five readings of the unattended automated office BP with the threshold for hypertension at least 135/85 mmHg. A total of 83% of study participants fell into the same groups in both analyses and their basic characteristics listed in Table 1 did not change substantially.

### Retinal parameters

**FIGURE 1:**
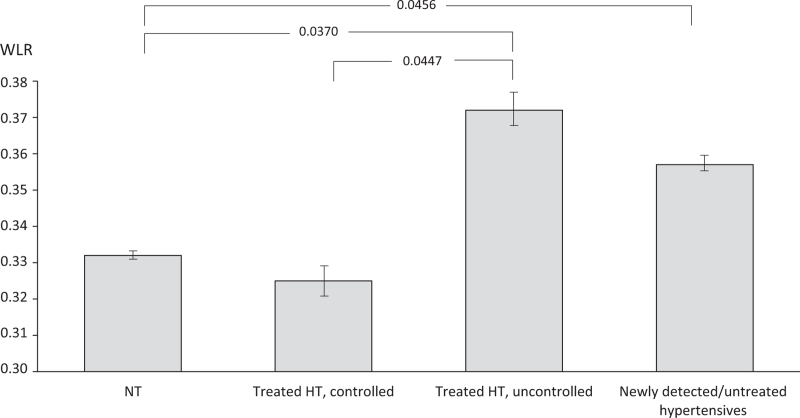
Wall-to-lumen ratio by study groups. Values are least square mean ± 95% CI, adjusted for age, sex, HbA1c, and triglycerides. HT, hypertensive individuals; NT, normotensive individuals; WLR, wall-to-lumen ratio.

Retinal parameters of the four groups based on office BP are summarized in Table 2. There was a tendency towards higher wall thickness in treated uncontrolled hypertensives compared to normotensive individuals and treated controlled hypertensive individuals. The WLR was significantly increased in treated uncontrolled hypertensive patients, as well as in individuals with newly detected, thus untreated hypertension (Fig. 1). There was no difference in WLR in treated and controlled hypertensive patients compared to normotensive individuals. The results were adjusted for age, sex, HbA1c, and triglycerides, which are the parameters thought to affect retinal microcirculation. The unadjusted analysis showed approximately the same results.

**TABLE 2:** Retinal parameters by study groups

	Normotensive individuals	Treated hypertensive individuals, controlled	Treated hypertensive individuals, uncontrolled	Newly detected/ untreated hypertensive individuals	*P*
JRCF, adj.^a^ (AU)	271 ± 5.3	274 ± 13.9	282 ± 15.1	264 ± 8.9	n.s.
VD, adj.^a^ (μm)	103.6 ± 0.94	105.8 ± 2.46	109.1 ± 2.68	104.2 ± 1.58	n.s.
LD, adj.^a^ (μm)	77.9 ± 0.61	79.9 ± 1.58	79.3 ± 1.72	77.0 ± 1.01	n.s.
WT, adj.^a^ (μm)	12.9 ± 0.3	12.9 ± 0.7	14.9 ± 0.7	13.6 ± 0.4	0.0675
WLR, adj.^a^	0.332 ± 0.006	0.325 ± 0.016	0.372 ± 0.018	0.357 ± 0.010	0.0458
WCSA, adj.^a^ (μm)	3769 ± 103	3886 ± 267	4546 ± 292	3945 ± 172	n.s.
RVR, adj.^a^ (mmHg/AU)	0.368 ± 0.008	0.366 ± 0.020	0.395 ± 0.022	0.442 ± 0.013	< 0.0001

Values are expressed as least square mean ± SE; ANCOVA. AU, arbitrary unit; JRCF, juxtapapilary retinal capillary blood flow; LD, luminal diameter; RVR, retinal vascular resistance; VD, vessel diameter; WCSA, wall cross-sectional area; WLR, wall-to-lumen ratio; WT, wall thickness.

a Adjusted for age, sex, HbA1c, and triglycerides.

Using the group allocation based on the automated office BP, the WLR was significantly increased in newly detected/untreated hypertensive individuals (*P* < 0.0314 vs. normotensive individuals). The two-way ANOVA (treatment Yes/No; BP controlled/uncontrolled) showed that WLR is closely related only to BP (*P* = 0.005).

## DISCUSSION

The relatively easy and noninvasive accessibility of retinal vessels may contribute to the understanding of pathological processes involved in cardiovascular disease.

### Principal findings

In this population-based study conducted in Whites, we found that WLR was increased in individuals with newly detected hypertension and in untreated hypertensive patients, compared to normotensive individuals or treated but controlled hypertensive patients. As WLR possibly reflects early vascular damage, our findings may support early initiation of antihypertensive treatment. Moreover, there was also no difference in WCSA, thus indicating a predominant eutrophic remodeling in this initial stage of hypertension [12]. Similarly, in our study population, there was also a tendency toward higher wall thickness in treated, uncontrolled hypertensive individuals and in newly detected/untreated hypertensive individuals compared to normotensive individuals and treated, controlled hypertensive individuals.

The group of treated but uncontrolled hypertensive patients and that of newly detected/untreated hypertensive individuals had the highest BP values both for office BP and for unattended automated office BP. A further analysis (two-way ANOVA) showed that WLR is closely related to BP, but not associated with drug treatment.

The WLR was previously found to have a prognostic impact in patients with hypertension and cerebrovascular disease [11]. There was also a positive association with urinary albumin excretion [17].

We would like to point out that in our study the examined retinal parameters of treated and controlled hypertensive patients did not differ from normotensive individuals. This finding may indicate that antihypertensive treatment in this subgroup was initiated in a timely manner before HMOD developed or, if changes in retinal microvasculature already existed, they might have regressed due to antihypertensive treatment.

Using optical coherence tomography angiography, a method mostly used for the detection of sight-threatening retinal disease, Hua *et al.*[18] found that hypertensive patients with standard and poor BP control had lower retinal vessel density in some of the retinal regions. On the contrary, similar to our study, the findings of intensively treated hypertensive patients did not differ from normotensive controls [18].

The regression of microvascular changes was so far documented only in isolated subcutaneous arterioles and small arteries [19]. As a close correlation between media-to-lumen ratio of subcutaneous small arteries and WLR of retinal arterioles was found, we may extrapolate that regression of changes in retinal arterioles is also possible [20].

### Study strength

The results were obtained from randomly selected individuals within the Czech post-MONICA study and are considered at least representative of the Czech urban population [14]. The same population was used to establish reference values of retinal microcirculation parameters [21].

### Limitations

The assessment of WLR of retinal arterioles using SLDF with AFFPIA has some limitations due to its in-vivo measurement, making it impossible to distinguish between functional (i.e. vasoconstriction) and structural changes. On the contrary, the examination of isolated subcutaneous arterioles and small arteries is performed in fully relaxed vessels [22].

The results of our study should be interpreted within the context of its strengths and limitations. As the study is a cross-sectional one, it has provided no data on longitudinal changes of retinal microcirculatory parameters with aging and related risk factors. Thus, there is an urgent need to initiate a follow-up study.

## CONCLUSION

Our results show that an increased WLR, reflecting early vascular damage, was found in newly detected individuals with hypertension and in untreated hypertensive patients, reflecting early hypertension mediated vascular damage. This observation supports early initiation of antihypertensive treatment to prevent further vascular remodeling and reverse it to the level of normotensive individuals. Our study also shows that a sophisticated examination of retinal microcirculation is feasible in epidemiological studies.

## ACKNOWLEDGEMENTS

The study was supported by grant 15-27109A provided by the Ministry of Health of the Czech Republic.

Renata Cífková: Conceptualization, Methodology, Funding acquisition, Resources, Project administration, Supervision, Validation, Formal analysis, Writing – original draft.

Joanna M. Harazny: Conceptualization, Methodology, Resources, Investigation, Supervision, Validation, Formal analysis, Writing – review and editing.

Jan Bruthans: Conceptualization, Methodology, Project administration, Investigation, Supervision, Writing – review and editing.

Peter Wohlfahrt: Conceptualization, Methodology, Investigation, Supervision, Validation, Formal analysis, Writing – original draft.

Alena Hrubeš Krajčoviechová: Conceptualization, Methodology, Writing – review and editing.

Věra Lánská: Methodology, Data curation, Formal analysis, Software, Validation, Writing – review and editing.

Július Gelžinský: Investigation, Validation, Writing – review and editing.

Markéta Mateřánková: Investigation, Validation, Writing – review and editing.

Štěpán Mareš: Investigation, Validation, Writing – review and editing.

Jan Filipovský: Conceptualization, Methodology, Resources, Project administration, Supervision, Validation, Formal analysis, Writing – review and editing.

Otto Mayer Jr: Conceptualization, Methodology, Resources, Project administration, Supervision, Validation, Formal analysis, Writing – review and editing.

Roland E. Schmieder: Conceptualization, Methodology, Resources, Supervision, Validation, Formal analysis, Writing – review and editing.

### Conflicts of interest

None.
